# Supplementary material for: Isobaric crosslinking mass spectrometry technology for studying conformational and structural changes in proteins and complexes
Source: eLife. 2024 Nov 14;13:RP99809. doi: 10.7554/eLife.99809 (PMC11563578; doi:10.7554/eLife.99809)
Supplement: Figure 1—figure supplement 2—source data 1. [file elife-99809-fig1-figsupp2-data1.zip › Figure 1-Figure supplement 2-source data 2.pdf]

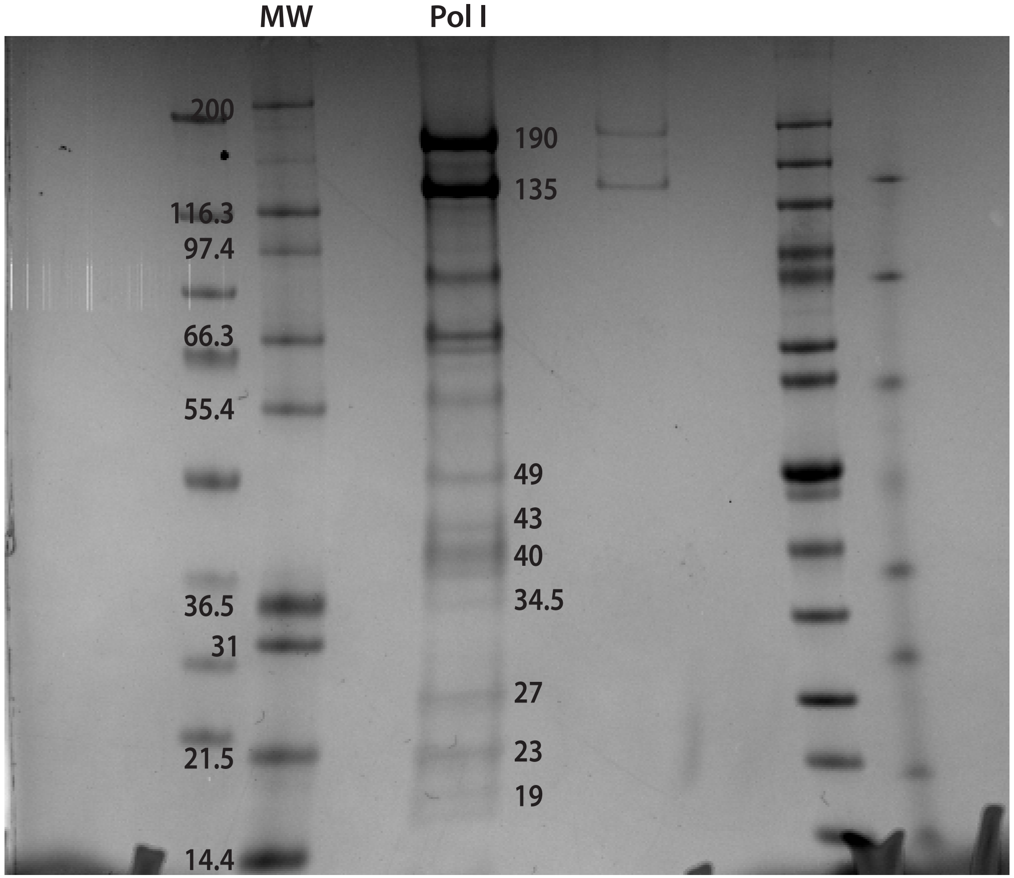

Figure1- Figure supplement 2- source data 1. The original gel for Figure 1-Figure supplement 2, indicating the relevant samples, bands and molecular weight marker sizes.
