# Supplementary figures and images for: Isobaric crosslinking mass spectrometry technology for studying conformational and structural changes in proteins and complexes
Source: eLife. 2024 Nov 14;13:RP99809. doi: 10.7554/eLife.99809 (PMC11563578; doi:10.7554/eLife.99809)

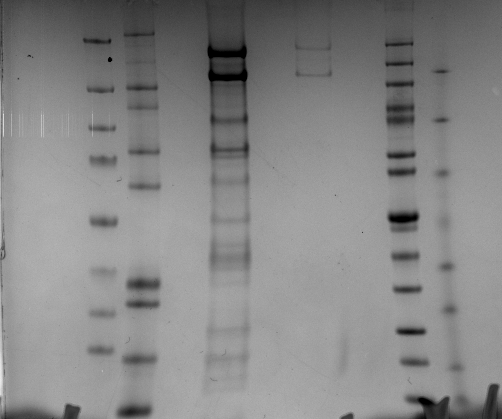

Supplement: Figure 1—figure supplement 2—source data 2. [file elife-99809-fig1-figsupp2-data2.zip › Figure 1-Figure supplent 2-source data 1.png]

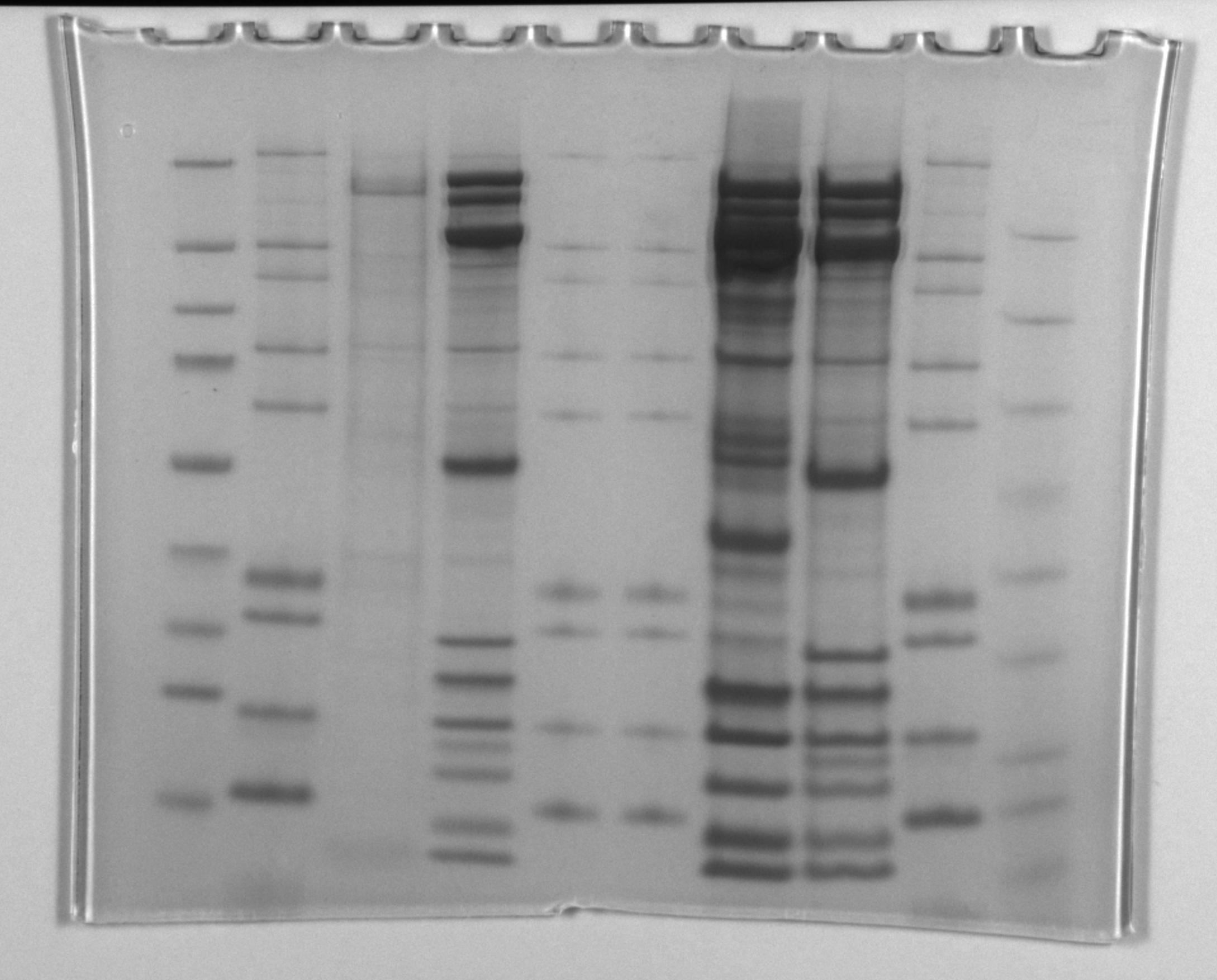

Supplement: Figure 4—source data 2. [file elife-99809-fig4-data2.zip › Figure 4 -source data 1.tif]
