# Supplementary material for: Isobaric crosslinking mass spectrometry technology for studying conformational and structural changes in proteins and complexes
Source: eLife. 2024 Nov 14;13:RP99809. doi: 10.7554/eLife.99809 (PMC11563578; doi:10.7554/eLife.99809)
Supplement: Figure 4—source data 1. [file elife-99809-fig4-data1.zip › Figure 4-source data 1.pdf]

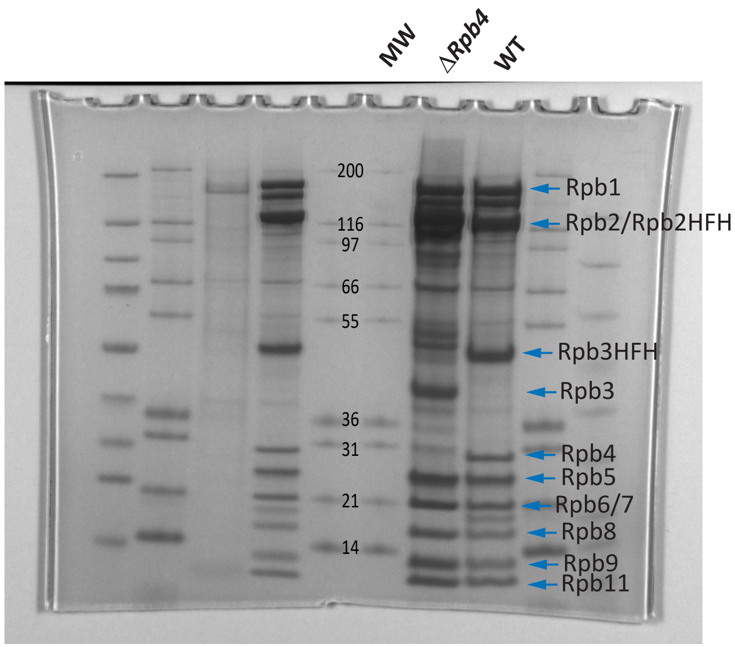

Figure 4- source data 1. The orginal gel for Figure 4a, indicating the relevant samples, bands, and molecular weight marker sizes.
